# Proposal of predictive model on survival in unresectable pancreatic cancer receiving systemic chemotherapy

**DOI:** 10.7150/jca.38861

**Published:** 2020-01-01

**Authors:** Noriko Ishii, Hiroki Nishikawa, Yoshinori Iwata, Hirayuki Enomoto, Hironori Tanaka, Nobuyuki Katakami, Takashi Nishimura, Hiroko Iijima, Shuhei Nishiguchi

**Affiliations:** 1 Division of Hepatobiliary and Pancreatic disease, Department of Internal Medicine, Hyogo College of Medicine, Nishinomiya, Hyogo, Japan.; 2 Department of gastroenterology, Takarazuka municipal hospital, Takarazuka, Hyogo, Japan.; 3 Department of oncology, Takarazuka municipal hospital, Takarazuka, Hyogo, Japan.

**Keywords:** Unresectable pancreatic cancer, Chemotherapy, Survival, Predictive model, Validation.

## Abstract

**Aims:** To construct a predictive model for overall survival (OS) in unresectable pancreatic cancer (PaC) undergoing systemic chemotherapy and to confirm its accuracy in an independent cohort.

**Patients and methods:** The training set (Ts) and the validation set (Vs) included 93 patients (median age=71 years) and 75 patients (median age=76 years). In the Ts, we examined variables linked to OS by uni- and multivariate analyses and constructed a predictive model for OS. Next, we evaluated the reproducibility of the proposed model in the Vs.

**Results:** In the multivariate analysis for the Ts, PaC stage IV (*P*=0.0020) and carbohydrate antigen (CA) 19-9 ≥437.5 IU/l (*P*=0.0237) were identified to be significant factors associated with OS. Patients with PaC stage IV or not were given a score of 1 or 0, whereas patients with CA19-9 ≥437.5 IU/l or <437.5 IU/l were given a score of 1 or 0. Sum of the point of PaC stage (0 or 1) and CA19-9 (0 or 1) was defined as “PaC-CA score”. In the Ts, there were 16 patients with score 0, 40 with score 1 and 37 with score 2, while in the Vs, there were 9 patients with score 0, 32 with score 1 and 34 with score 2. Overall *P* values reached significance in the Ts (*P*=0.0002), the Vs (*P*=0.0029) and the combined Ts and Vs (*P*<0.0001) among patients with PaC score 0, 1 and 2.

**Conclusion:** PaC-CA score can be helpful for risk stratification in PaC patients undergoing systemic chemotherapy.

## Introduction

Pancreatic cancer (PaC) is associated with a dismal prognosis, highlighted by the close relationship between cancer incidence and mortality 1-3. The 5-year overall survival (OS) rate in PaC patients with advanced tumor status is extremely low 1-3. The dismal prognosis is attributed to several causes: most importantly, numerous PaC patients are found in an advanced condition and the tumor is often unresectable because of its invasion to major vessels or distant metastases although several approaches to diagnosing PaC in its early stages have been attempted 1, 2, 4-8. According to Japanese cancer statistical data in 2016, PaC has the seventh-highest rate of incidence and it is the fourth leading cause of death in Japan 9. The Japanese clinical practice guidelines for systemic chemotherapy in unresectable PaC patients recommend the administration of following chemotherapeutic drugs: gemcitabine monotherapy, S-1 monotherapy, combined gemcitabine and S-1 therapy, combined nab-paclitaxel and gemcitabine therapy, or a combination chemotherapeutic regimen consisting of irinotecan, fluorouracil, oxaliplatin and leucovorin, considering baseline characteristics such as age, organ function, performance status (PS) and tumor status of each patient 10-15.

To predict prognosis in unresectable PaC patients undergoing systemic chemotherapy may be meaningful because it may be involved in clinical decision making whether to continue the treatment or not. Appropriate predictive models can offer a simple alternative in clinical sequences. We anticipate that a simple predictive model for OS in unresectable PaC patients will be useful in our daily clinical practice. However, to the best of our knowledge, there have been few reports regarding predictive models in unresectable PaC patients undergoing systemic chemotherapy, although numerous predictive models in PaC patients undergoing surgery has been reported 16-25. The goal of the current study is to construct a simple predictive model for OS in unresectable PaC patients treated with systemic chemotherapy and to confirm its accuracy in an independent cohort.

## Patients and methods

### Patients

Between June 2008 and May 2018, a total of 97 patients with unresectable systemic chemotherapy naïve PaC patients were admitted at Hyogo College of Medicine Hospital (Division of Hepatobiliary and Pancreatic disease, Department of Internal Medicine), Hyogo, Japan. Four patients with lost to follow-up or insufficient clinical data were excluded from the analysis. Thus, a total of 93 PaC patients were analyzed (the training set (Ts)). On the other hand, between July 2007 and January 2019, a total of 82 patients with unresectable systemic chemotherapy naïve PaC patients were admitted at the Division of Gastroenterology, Takarazuka municipal hospital, Hyogo, Japan. Seven patients with lost to follow-up or insufficient clinical data were also excluded from the analysis. A total of 75 PaC patients were therefore analyzed (the validation set (Vs)).

### Diagnosis for PaC

PaC was diagnosed primarily according to the current guidelines 26. In brief, abdominal ultrasonography and dynamic computed tomography (CT) of the whole pancreas was routinely performed before starting systemic chemotherapy. In cases without typical radiological tumor findings for PaC, tumor biopsy or endoscopic ultrasonography-guided fine needle aspiration was in consideration 27. In this study, the pathological diagnosis was confirmed in 44 cases (47%) in the Ts and 54 cases (66%) in the Vs.

### Systemic chemotherapy for PaC

Each attending physician determined chemotherapeutic agents through discussion with colleagues. In PaC subjects without remarkable risk factors, the recommended starting dose of each chemotherapeutic drug (S-1, gemcitabine, 5-fluorouracil or nab-paclitaxel) was administered 4, 28. The reduced starting dose was administered to some patients based on clinical characteristics, including age, ECOG-PS, body weight, and laboratory data. During systemic chemotherapy, the dose of chemotherapeutic drugs was appropriately adjusted by each attending physician considering the severity of adverse events. In patients with adverse events, systemic chemotherapy was stopped until the clinical symptoms improved to grade 1 or 2, and other alternative treatment regimens were in consideration. Other alternative regimens were also in consideration for subjects with poor treatment response to initial chemotherapy. Systemic chemotherapy was continued until any of the following conditions occurred: unacceptable toxicity for chemotherapy, tumor progression or the patient's wish to stop chemotherapy.

### Evaluation for treatment response

Principally, evaluation for the treatment response for systemic chemotherapy was done every 2-4 months following the start of chemotherapy, based on the Response Evaluation Criteria in Solid Tumors (RECIST ver. 1.1) using radiological findings and tumor markers 29. The most favorable treatment response was determined using the following four classifications: (i) Complete response (CR); (ii) partial response (PR); (iii) stable disease (SD); (iv) progressive disease (PD). The proportion of patients with the best treatment response rates of CR or PR was regarded as the objective tumor response rate (ORR), while that of patients with the best treatment response rates of CR, PR or SD was regarded as the disease control rate (DCR).

### Our study plan

**Figure 1:**
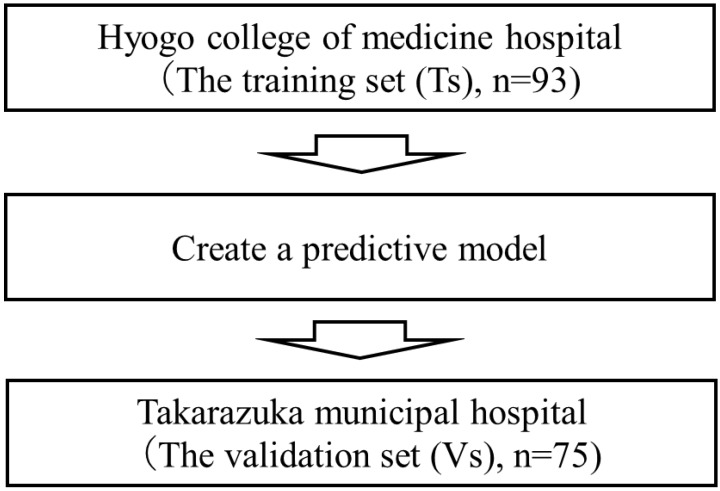
Study flow chart.

For the aim of our study, a Ts in whom systemic chemotherapy was performed in Hyogo college of medicine hospital (n=93) was formed. In the Ts, we examined variables linked to OS by uni- and multivariate analyses and constructed a predictive model for predicting OS. Next, we evaluated the reproducibility of the proposed model in the subsequent Vs (n=75), which included patients in the other hospital (Takarazuka municipal hospital). (Figure 1) Clinical data in the Ts were examined retrospectively and the constructed model was also verified in a retrospective manner for the independent Vs. Psoas muscle index (PMI) for the assessment of muscle mass was measured using CT as reported elsewhere 30.

Institutional review boards in each participating hospital approved the current study protocol (approval no. 2117 in Hyogo college of medicine hospital and 201707 in Takarazuka municipal hospital), with strict compliance for all of the provisions of the Declaration of Helsinki.

### Statistical analysis

A simple predictive model on survival was built from subjects in the Ts and validated in separate, independent patients in the Vs. Firstly, as mentioned above; univariate analysis was conducted to identify candidate variables among several clinical parameters to create a predictive model. The median value for each parameter was selected in order to divide the study population into two groups, which was then treated as nominal variables in the univariate analysis. Parameters with *P*<0.05 in the univariate analysis were included in the multivariate Cox hazard model. Factors with *P*<0.05 in the multivariate analysis were finally chosen as components of the novel predictive model. Based on these multivariate predictors, our proposed model was created. In the subsequent Vs, we tested the diagnostic accuracy of the created model.

OS curve was built by the Kaplan-Meier method and compared by the log-rank test. In continuous variables, the statistical comparison among groups was done by Student's *t* test or Mann Whitney *U* test, as applicable. Categorical variables were compared by Fisher's exact tests or Pearson χ^2^ test, as applicable. Unless otherwise stated, data were expressed as median value (range). *P* value less than 0.05 was considered to be significant statistically with the JMP 14 (SAS Institute Inc., Cary, NC).

## Results

### Baseline data

The baseline data in the Ts (n=93) and the Vs (n=75) in this study were demonstrated in table 1. In comparison of the Ts and the Vs, in terms of age (*P*=0.0140), gender (*P*=0.0009), PS (*P*=0.0380), primary site (*P*=0.0011), prothrombin time (*P*=0.0002), carcinoembryonic antigen (*P*=0.0117) and carbohydrate antigen 19-9 (CA19-9, *P*=0.0170), the differences in the two groups reached significance. (Table 1) The median follow-up period in the Ts and the Vs were 255 days and 217 days, respectively. In terms of initial chemotherapeutic regimens, gemcitabine monotherapy was done in 57 patients, S-1 monotherapy in 12, combined gemcitabine and S-1 therapy in 3, combined nab-paclitaxel and gemcitabine therapy in 19, uracil and tegafur therapy in 1, and FOLFIRINOX in 1 in the Ts, while gemcitabine monotherapy was done in 44 patients, S-1 monotherapy in 8, combined gemcitabine and S-1 therapy in 6, combined nab-paclitaxel and gemcitabine therapy in 11, and FOLFIRINOX in 6 in the Vs.

### OS in the Ts and the Vs

**Figure 2:**
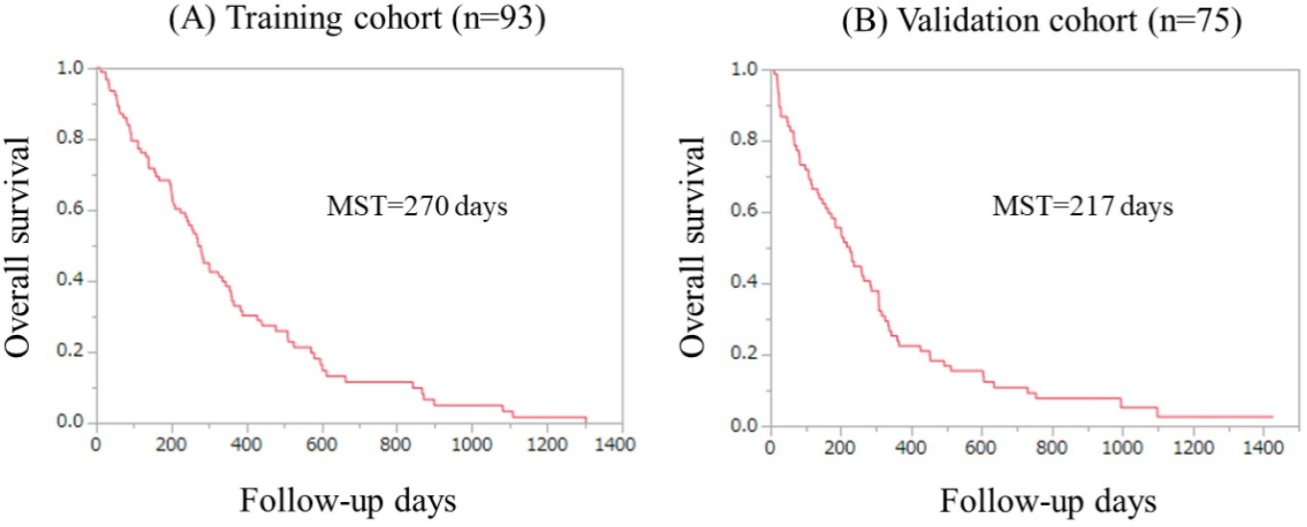
Overall survival in the Ts (A) and the Vs (B). MST indicates median survival time.

Kaplan-Meier curves in the Ts and the Vs were shown in figure 2A and 2B. The median survival time in the Ts and the Vs were 270 days and 217 days, respectively.

### Best tumor response during the follow-up period in the Ts and the Vs

In the Ts, regarding the best treatment response during chemotherapy, CR was achieved in 0, PR in 7, SD in 23, PD in 48 and not evaluated (NE) in 15 patients. The ORR and DCR were therefore 7.5% (7/93) and 32.3% (30/93), respectively. In the Vs, regarding the best treatment response during chemotherapy, CR was achieved in 0, PR in 4, SD in 19, PD in 40 and NE in 12 patients. The ORR and DCR were therefore 5.3% (4/75) and 30.7% (23/75), respectively.

### Causes of death

In the Ts, 79 (84.9%) patients succumbed during the observation period. All patients died because of the advanced PaC status. In the Vs, 70 (93.3%) patients died during the observation period. All patients died because of the advanced PaC status.

### Uni- and multivariate analyses of factors associated with OS in the Ts

Univariate analysis observed the following items as significantly associated with OS for the Ts: maximum tumor size ≥34 mm (*P*=0.0204); PaC stage IV (*P*=0.0015); and CA19-9 ≥437.5 IU/l (*P*=0.0061). (Table 2) The hazard ratios and 95% confidence intervals in the multivariate analysis for the three items with *P*<0.05 in the univariate analysis were presented in table 2. PaC stage IV (*P*=0.0020) and CA19-9 ≥437.5 IU/l (*P*=0.0237) were identified to be significant prognostic factors associated with OS.

### Our proposed predictive model

Based on the results of multivariate analysis, patients with PaC stage IV were allocated a score of 1, whereas patients with other PaC stage than stage IV were allocated a score of 0. Patients with CA19-9 ≥437.5 IU/l were allocated a score of 1, whereas patients with CA19-9 <437.5 IU/l were allocated a score of 0. Sum of the point of PaC stage (0 or 1) and CA19-9 (0 or 1) was defined as “PaC-CA score”. PaC-CA score therefore ranged from 0 to 2. (Table 3) We tested the predictive ability of PaC-CA score for survival in the Ts and the Vs. In the Ts, there were 16 patients with PaC-CA score 0, 40 with PaC-CA score 1 and 37 with PaC-CA score 2, while in the Vs, there were 9 patients with PaC-CA score 0, 32 with PaC-CA score 1 and 34 with PaC-CA score 2.

**Figure 3:**
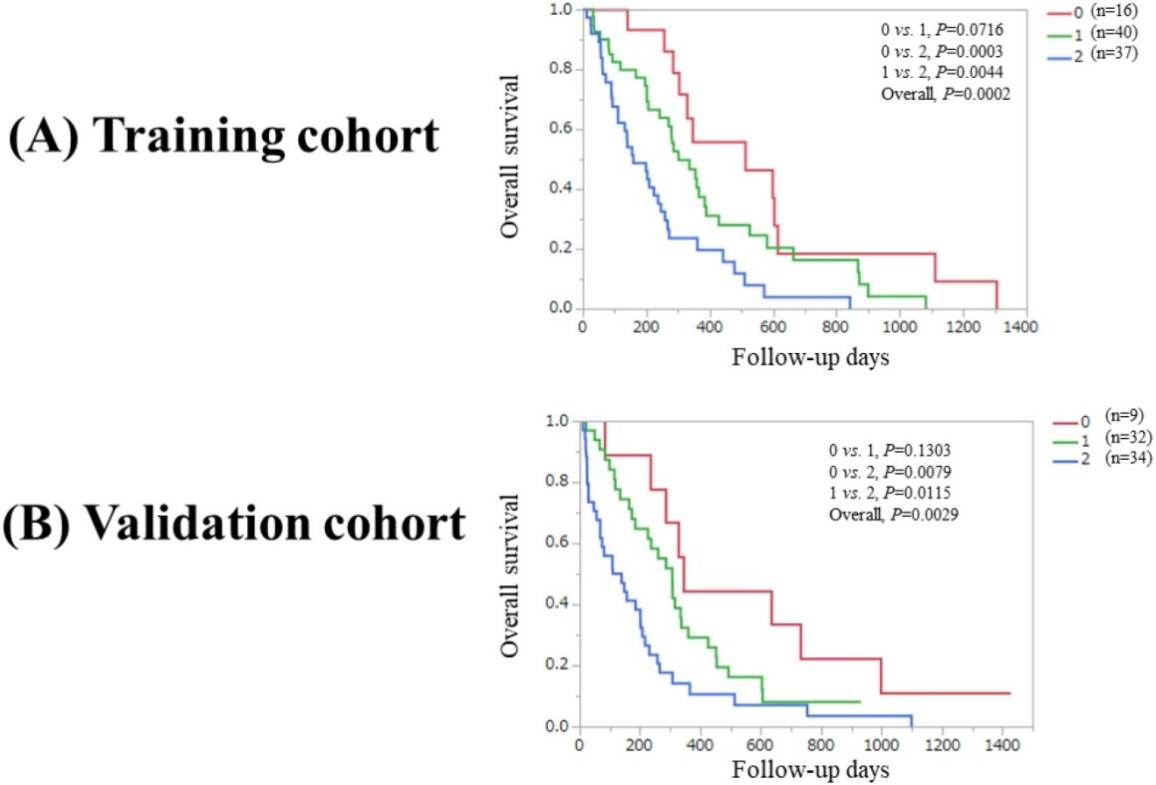
(A) Overall survival stratified by PaC-CA score (0, 1 and 2) in the Ts. (B) Overall survival stratified by PaC-CA score (0, 1 and 2) in the Vs. PaC-CA scoring system is our newly proposed predictive model consisting of pancreatic cancer stage and CA19-9 level.

Overall *P* value reached significance (*P*=0.0002) in the Ts among patients with PaC score 0, 1 and 2 (*P* values between each two group: 0 *vs.* 1, *P*=0.0716; 0 *vs.* 2, *P*=0.0003; and 1 *vs.* 2, *P*=0.0044). While overall *P* value reached significance (*P*=0.0029) in the Vs among patients with PaC score 0, 1 and 2 (*P* values between each two groups: 0 *vs.* 1, *P*=0.1303; 0 *vs.* 2, *P*=0.0079; and 1 *vs.* 2, *P*=0.0115). (Figure 3A and 3B).

**Figure 4:**
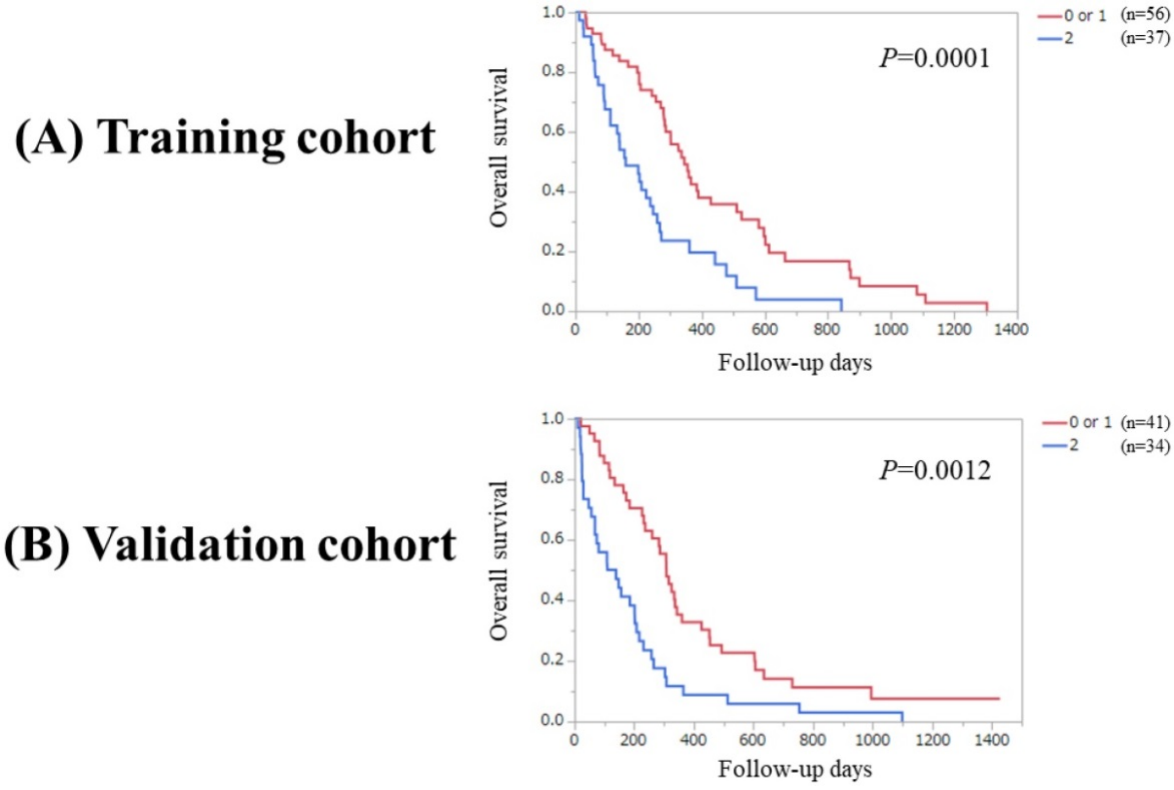
(A) Overall survival stratified by PaC-CA score (0 or 1 and 2) in the Ts. (B) Overall survival stratified by PaC-CA score (0 or 1 and 2) in the Vs.

**Figure 5:**
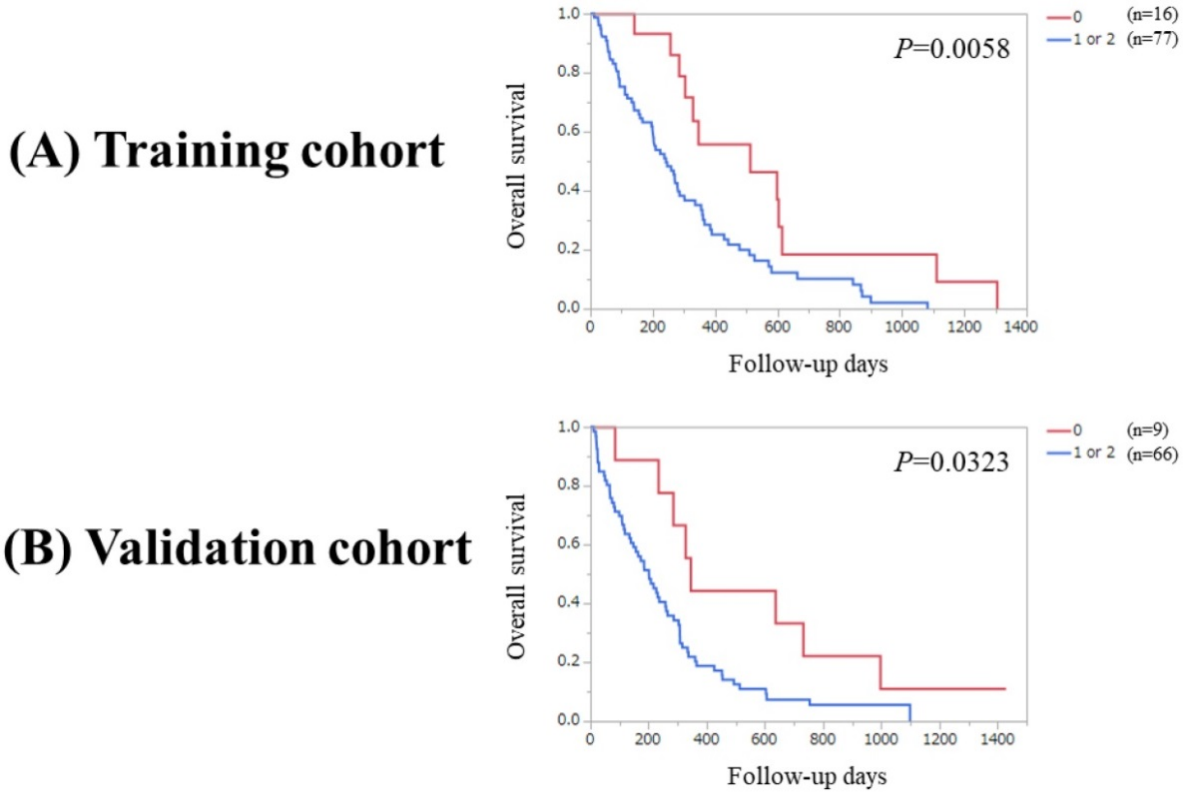
(A) Overall survival stratified by PaC-CA score (0 and 1 or 2) in the Ts. (B) Overall survival stratified by PaC-CA score (0 and 1 or 2) in the Vs.

Significant difference was observed between patients with PaC score 0 or 1 (n=56) and 2 in the Ts (*P*=0.0001). Likewise, significant difference was observed between patients with PaC score 0 or 1 (n=41) and 2 in the Vs (*P*=0.0012). (Figure 4A and 4B) Significant difference was noted between patients with PaC score 0 and 1 or 2 (n=77) in the Ts (*P*=0.0058). Similarly, significant difference was noted between patients with PaC score 0 and 1 or 2 (n=66) in the Vs (*P*=0.0323). (Figure 5A and 5B).

**Figure 6:**
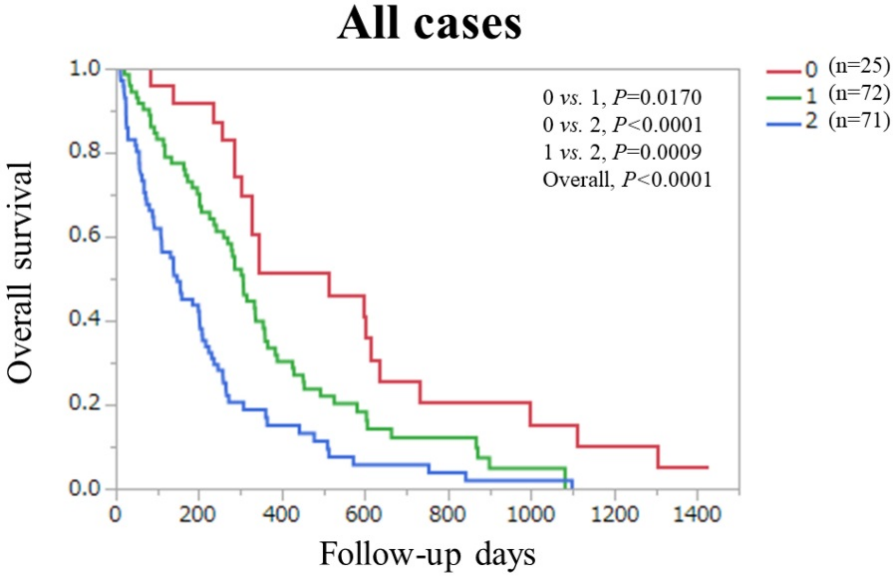
Overall survival stratified by PaC-CA score (0 and 1 or 2) in the combined Ts and Vs.

Finally, in the combined Ts and Vs (n=168), overall *P* value reached significance (*P*<0.0001) among patients with PaC score 0 (n=25), 1 (n=72) and 2 (n=71) (*P* values between each two group: 0 *vs.* 1, *P*=0.0170; 0 *vs.* 2, *P*<0.0001; and 1 *vs.* 2, *P*=0.0009). (Figure 6).

## Discussion

Decision making in the clinical settings in cancer patients may be challenging and predictive model may be helpful from the viewpoint of appropriate decision making. Here in the current study, we created a simple predictive model called “PaC-CA score”, which included tumor stage and CA19-9 level. Clinicians are very familiar with these parameters in the routine clinical practice and thus PaC-CA score may be convenient and easy to access. Several predictive models such as combined platelet-to-lymphocyte ratio and CA 19-9 were proposed for PaC patients undergoing surgery 16, 17, 19-23. While, to the best of our knowledge, this is the first study for constructing a simple predictive model for OS in unresectable PaC patients undergoing systemic chemotherapy, and validating its accuracy in an independent cohort, which was a major strong point of our current analysis.

In our results, the overall significance was noted in the Ts, the Vs and the combined Ts and Vs among patients with PaC-CA score 0, 1 and 2. Baseline characteristics between the Ts and the Vs were different in several parameters as shown in table 1 and our proposed model was well confirmed in the Vs and the combined Ts and Vs. These results denoted that our proposed predictive model can be helpful for risk stratification in PaC patients undergoing systemic chemotherapy. While between PaC-CA score 0 and 1 in the Ts and between PaC-CA score 0 and 1 in the Vs, significant difference was not noted. These results may be attributed to the small sample size of patients with PaC-CA score 0 in the Ts (n=16) and the Vs (n=9). In comparison of Kaplan-Meier curves between PaC-CA score 0 and 1, that of PaC score 0 is almost persistently above that of PaC score 1 as presented in figure 3, and thus we believe our predictive model is robust.

A recent study reported that CA19-9 expression in mice activated the epidermal growth factor receptor signaling, and it also cooperated with the *Kras* oncogene to develop aggressive pancreatic cancer 31. Takagi, et al. demonstrated that the elevation of post-operative serum CA19-9 value was associated with an adverse outcome and reflected positivity of resection margins, and high pre-operative CA19-9 values suggested the presence of occult distant metastasis in PaC patients undergoing surgery 32. Other several reports also demonstrated that CA19-9 may be involved in prognostic implication in PaC patients 16, 17, 33-35. Our results were in agreement with these reports.

Recently, sarcopenia as defined by low skeletal muscle mass and low skeletal muscle function has been gaining much interest due to its prognostic impact in cancer patients 36-39. A recent meta-analysis regarding outcome in PaC patients receiving surgery reported that sarcopenia was associated with increased peri-operative mortality and decreased OS 37. While contrary to our expectations, PMI was not selected as significant factor for OS in our cohort. In our data, PMI in male ranged from 0.703 to 8.011 cm^2^/m^2^ (median, 2.447 cm^2^/m^2^) and PMI in female ranged from 0.509 to 4.662 cm^2^/m^2^ (median, 1.952 cm^2^/m^2^), which is considerably lower compared with the cutoff values of PMI for muscle mass decrease in male (6.36 cm^2^/m^2^) and female (3.92 cm^2^/m^2^) reported by Hamaguchi, et al 40. These results may be associated with not only sarcopenia but also cancer-related cachexia 41, 42. The fact that most of our analyzed subjects had low PMI may explain for the non-significance of PMI on OS.

Several limitations warrant mention to this study. First, our current investigation was a retrospective observational study and biases inherent to retrospective studies were unable to be completely removed, although our proposed model was verified in an independent cohort. Second, the starting chemotherapeutic drugs differed between the analyzed subjects and these differences may lead to bias for OS. Third, the sample size was relatively small for analysis (less than 100 cases both in the Ts and the Vs), potentially creating bias. Finally, our study cohort only included Japanese unresectable PaC patients in whom baseline characteristics such as body weight are different from PaC patients in Western countries 43. Whether our current results are directly applied to different ethnic backgrounds is therefore unclear. However, the present study results demonstrated that PaC-CA score can be helpful for risk stratification in PaC patients treated with systemic chemotherapy.

In conclusion, our proposed predictive model can be useful in unresectable PaC patients undergoing systemic chemotherapy.

## Figures and Tables

**Table 1:** Baseline characteristics in the training set and validation set.

Variables	Training set (N=93)	Validation set (N=75)	*P* value
Age (years)	71 (39-89)	76 (46-91)	0.0140
Gender, male / female	44 / 49	55 / 20	0.0009
Performance status, 0 / 1 or 2 / unknown	75 / 18 / 0	61 / 12 / 2	0.0380
Body mass index (kg/m^2^)	21 (15.1-33.1)	20.8 (14.9-32.6)	0.6385
Pancreatic cancer stage, IV / others	67 / 26	56 /19	0.7293
Maximum tumor size (cm)	33 (9-83)	35 (16-110)	0.2203
Primary site, uncus or head / body or tail	58 / 35	27 / 48	0.0011
Total bilirubin (mg/dL)	0.8 (0.3-6.6)	0.75 (0.3-15.8)	0.6109
Serum albumin (g/dL)	3.5 (1.8-4.6)	3.6 (2-4.8)	0.1252
Prothrombin time (%)	86.2 (43.5-127)	78 (30-116)	0.0002
Platelet count (×10^4^/mm^3^)	20.8 (7.1-61)	19.5 (8-49.3)	0.1350
White blood cell (×10^3^/μl)	6.11 (2.54-29.76)	6.36 (2.43-36.24)	0.1274
Hemoglobin (g/dl)	11.7 (7.5-15.7)	11.9 (1.4-17.3)	0.7897
Serum creatinine (mg/dl)	0.65 (0.28-7.41)	0.77 (0.43-1.53)	0.4275
C reactive protein (mg/dl)	0.6 (0-22.0)	0.8 (0-22.51)	0.0888
AST (IU/L)	26 (11-265)	22 (2-149)	0.7497
ALT (IU/L)	29 (6-289)	23 (6-294)	0.5417
ALP (IU/L)	361 (119-1982)	365 (131-2104)	0.5326
GGT (IU/L)	100 (11-1023)	89 (14-1315)	0.2586
Amylase (IU/L)	63.5 (7-357)	66.5 (23-583)	0.3758
CEA (IU/L)	4.25 (1.2-286.1)	7.7 (0.8-710)	0.0117
CA19-9 (IU/L)	437.5 (0.6-42414)	1344 (0.2-500000)	0.0170
Psoas muscle index (cm^2^/m^2^, male)	2.576 (1.340-5.708)	2.125 (0.703-8.011)	0.0788
Psoas muscle index (cm^2^/m^2^, female)	1.947 (0.509-4.662)	1.890 (0.955-3.212)	0.7126

Data are expressed as number or median value (range). AST; aspartate aminotransferase, ALT; alanine aminotransferase, ALP; alkaline phosphatase, GGT; gamma glutamyl transpeptidase, CEA; carcinoembryonic antigen, CA19-9; carbohydrate antigen 19-9.

**Table 2:** Uni- and multivariate analyses of factors linked to overall survival in the training set.

Variables	Number	Univariate	Multivariate		
		*P* value	HR	95% CI	*P* value
Gender, male/female	44/49	0.2058			
Psoas muscle index (cm^2^/m^2^) decrease, yes/no	43/45	0.8079			
Age ≥71 years, yes/no	48/45	0.4562			
Body mass index >20.95 kg/m^2^, yes/no	47/46	0.8414			
White blood cell count ≥6110×10^3^/μl, yes/no	47/46	0.6168			
Performance status 0, yes/no	75/18	0.2208			
Maximum tumor size ≥34mm, yes/no	46/47	0.0204	1.403	0.885-2.232	0.1489
Tumor stage IV, yes/no	67/26	0.0015	2.256	1.332-4.029	0.0020
CA19-9 ≥437.5 IU/l, yes/no	47/46	0.0061	1.715	1.075-2.757	0.0237
Serum albumin ≥3.6 g/dl, yes/no	43/50	0.2275			
Platelet count ≥20.9×10^4^/mm^3^, yes/no	46/47	0.0910			
Prothrombin time ≥86.2%, yes/no	47/46	0.0735			
C reactive protein ≥0.6 mg/dl, yes/no	46/47	0.0750			

CA19-9; carbohydrate antigen 19-9, HR; hazard ratio, CI; confidence interval.

**Table 3:** Our proposed predictive model, called “PaC-CA score”.

	Point		
Pancreatic cancer stage	Stage IV	Others	
	1	0	
CA19-9	≥437.5 IU/L	<437.5 IU/L	
	1	0	
PaC-CA score	2	1	0

PaC; pancreatic cancer, CA; carbohydrate antigen.

## Abbreviations

PaC: Pancreatic cancer
OS: overall survival
CT: computed tomography
PS: performance status
RECIST: Response Evaluation Criteria in Solid Tumors
CEA: carcinoembryonic antigen
CA19-9: carbohydrate antigen 19-9
CR: complete response
PR: partial response
SD: stable disease
PD: progressive disease
ORR: objective response rate
DCR: disease control rate
NE: not evaluated
Ts: training set
Vs: validation set
PMI: psoas muscle index
